# A CDE-based data structure for radiotherapeutic decision-making in breast cancer

**DOI:** 10.1186/s12911-025-03036-1

**Published:** 2025-07-01

**Authors:** Fabio Dennstädt, Maximilian Schmalfuss, Johannes Zink, Janna Hastings, Roberto Gaio, Max Schmerder, Nikola Cihoric, Paul Martin Putora

**Affiliations:** 1 Department of Radiation Oncology, HOCH Cantonal Hospital St. Gallen, St. Gallen, Switzerland; 2 Department of Radiation Oncology, Inselspital, Bern University Hospital and University of Bern, Bern, Switzerland; 3 Department of Radiology, HOCH Cantonal Hospital St. Gallen, St. Gallen, Switzerland; 4 Institute for Computer Science, University of Würzburg, Würzburg, Germany; 5 School of Medicine, University of St. Gallen, St. Gallen, Switzerland; 6 Institute for Implementation Science in Health Care, University of Zurich, Zurich, Switzerland; 7 Swiss Institute of Bioinformatics, Lausanne, Switzerland

**Keywords:** Data systems, Radiation oncology, Common data element, Breast neoplasms, Decision making

## Abstract

**Background:**

The growing complexity of oncology and radiation therapy demands structured and precise data management strategies. The National Institutes of Health (NIH) have introduced Common Data Elements (CDEs) as a uniform approach to facilitate consistent data collection. However, there is currently a lack of a comprehensive set of CDEs for describing situations for and within radiation oncology. Specifically for breast cancer, where radiotherapeutic decision-making is complex and based on multiple diverse criteria, there is a clear need for more standardized data. Aim of this study was to create a CDE-based data structure for radiotherapeutic decision-making in breast cancer to promote structured data collection on the level of a local hospital.

**Methods:**

Between May 2023 and May 2024, we conducted a case study at the radiation therapy department of a local hospital to develop a CDE-based data structure for radiotherapeutic decision-making in breast cancer. Local Standard Operating Procedures (SOPs) were analyzed to identify relevant decision-making criteria used in clinical practice. Corresponding CDEs were identified, and a structured data framework based on these CDEs was created. The framework was translated into machine-readable JavaScript Object Notation (JSON) format. Six clinical practice guidelines of the American Society for Radiation Oncology (ASTRO) were analyzed as full text to evaluate how many guideline recommendations and corresponding decision-making criteria could be represented using our framework.

**Results:**

We identified 31 decision-making criteria from local SOPs, formalized into 46 CDEs. A hierarchical structure within an object-oriented data framework was created and converted into JSON format. 94 recommendations with mentioning of decision-making criteria in 216 cases were identified across the six ASTRO guidelines. In 151 cases (70.0%) the mentioned criterion could be presented with the data framework.

**Conclusions:**

The CDE-based data structure provides a standardized, machine-readable framework for documenting and exchanging radiotherapeutic decision-making data in breast cancer patients. While further refinement is needed for broader interoperability, this approach facilitates structured data collection, enhances IT integration and supports standardized communication across different stakeholders.

**Supplementary Information:**

The online version contains supplementary material available at 10.1186/s12911-025-03036-1.

## Introduction

The application of modern information technology (IT) systems and artificial intelligence (AI) is of ever-increasing relevance in modern oncology and radiation oncology. For management of medical data within databases and for data processing within most IT applications, it is necessary to store the data in a structured and precise way. The concept of common data elements (CDEs), introduced in 2011 by the National Institutes of Health (NIH) for consistent data collection in clinical trials, can be used for this purpose as it facilitates clear definitions of semantic information. As explained in the definition, “a common data element is a standardized, precisely defined question, paired with a set of allowable responses, used systematically across different sites, studies, or clinical trials to ensure consistent data collection” [1, 2].

While the concept of CDEs is already further advanced in medical disciplines like radiology [3] or neurology [4], currently there are only few defined data elements for radiation oncology [5]. However, structured documentation using this concept would be of high value in a technical discipline like radiation oncology – not only for research, but also for management of real-world data (RWD). The International Society for Radiation Oncology Informatics (ISROI) is therefore actively working on promoting the usage of CDEs in radiation oncology [6].

For clinical decision-making international guidelines as well as locally defined standard operating procedures (SOPs) are fundamental. While guidelines, SOPs and other sorts of literature regarding clinical decision-making are mentioning some sort of decision-making criteria, those are usually not presented in a formalized and clearly defined way [7]. The clinical decision-making in breast cancer patients is particularly complex, as multiple factors and criteria are relevant while decision-making becomes more personalized and tailored to individual circumstances. As a result, a variety of IT-based tools and clinical decision-support systems (CDSS) are being developed to help clinicians in complex situations [8]. Unfortunately, there is no common semantic structure defined, which would facilitate precise and structured documentation and communication of the data representing a breast cancer situation.

### Using CDEs for data collection in clinical practice and for Real-World data

While primarily developed for standardized data collection within clinical trials, CDEs are increasingly being used for data management in daily clinical life [9]. In practice, the concept of CDEs can be applied to various media for documentation of medical data, including case report forms (CRF), electronic health records (EHR), clinical information systems (CIS) or analog document forms [10, 11]. This idea of using CDEs for documentation in daily clinical life becomes of additional interest regarding the increasing interest in RWD for addressing research questions while supporting daily healthcare [9].

CDEs provide a common language for enabling structural and semantic interoperability. They allow for the alignment of data from various sources like EHR, healthcare claims, and patient-generated data streams. By expressing CDEs in machine-computable formats, data can be mapped, transformed, and combined across disparate sources [12].

### Providing semantic information using CDEs

CDEs are a powerful yet simple concept that focus on the meaning of medical information [13]. If used within an appropriate framework, they can be used to clearly present the semantic data of a medical situation.

Several initiatives to define structured breast cancer data (for both research and clinical practice) have previously been conducted. Key initiatives include the National Cancer Institute’s (NCI) Breast Oncology Local Disease (BOLD) Task Force [14], which developed CRFs according to the CDE concept for localized breast cancer trials. The successful application for RWD collection was demonstrated by Villarreal-Garza et al. in the Joven & Fuerte Prospective cohort for Mexican breast cancer patients [15]. Additional studies have shown the feasibility and added value of structured RWD collection in oncology generally and specifically for breast cancer [16]. There furthermore have been efforts made regarding definition and clarification of CDEs in the domain of breast cancer for some research registries [17]. Nevertheless, there is still no broader framework for structured documentation of breast cancer data used commonly among different stakeholders (particularly not in clinical practice) [18]. At the same time, there are corresponding semantic concepts and data structures used within the logic of CIS, forms and clinical documents [5, 19].

Previous initiatives primarily followed a “top-down” approach, developing new abstract data models intended as guidelines for proper data collection. While this facilitates clear documentation in clinical trials, it also enforces new documentation practices on healthcare facilities, including additional administrative burden for precise data collection.

### Objective and relevance of the study

This work aims to take a different approach resembling more a “bottom-up” methodology—not prescribing new data models, but instead using the CDE concept aligned with existing local documentation practices and focusing on information relevant for clinical decision-making. By collecting RWD using CDEs, yet without enforcing new documentation standards, the benefits of the concept can be maintained without the administrative overhead present in clinical trials. Successful application of such an approach would represent a crucial starting point for achieving interoperability between various stakeholders by breaking down the clinical information in given real world documents into the smallest semantic units in the form of CDEs.

We aimed to create a data structure based on radiotherapeutic decision-making criteria used in clinical practice for breast cancer. By using the CDE concept, it is possible to create a universal data input layer for any further system of decision-making afterwards, including software systems as well as human evaluators.

## Methods

### Study design and setting

We conducted a case study at the radiation therapy department of the Kantonsspital St. Gallen (Switzerland) developing a CDE-based data structure based on the local SOPs on radiation therapy of breast cancer. These SOPs are used in daily clinical life by the clinicians of our research team and cover various breast cancer situations providing treatment recommendations about indication, radiation therapy planning, dose prescription etc. While these SOPs represent the standards for our local department, they are in large parts influenced by the recommendations of the St. Gallen International Breast Cancer Consensus Conference [20].

The study was conducted by an interdisciplinary expert panel consisting of clinicians, computer scientists and healthcare informaticians between May 2023 and May 2024.

### Identifying relevant CDEs used in clinical Decision-Making for breast Cancer patients to undergo radiation therapy

**Fig. 1:**
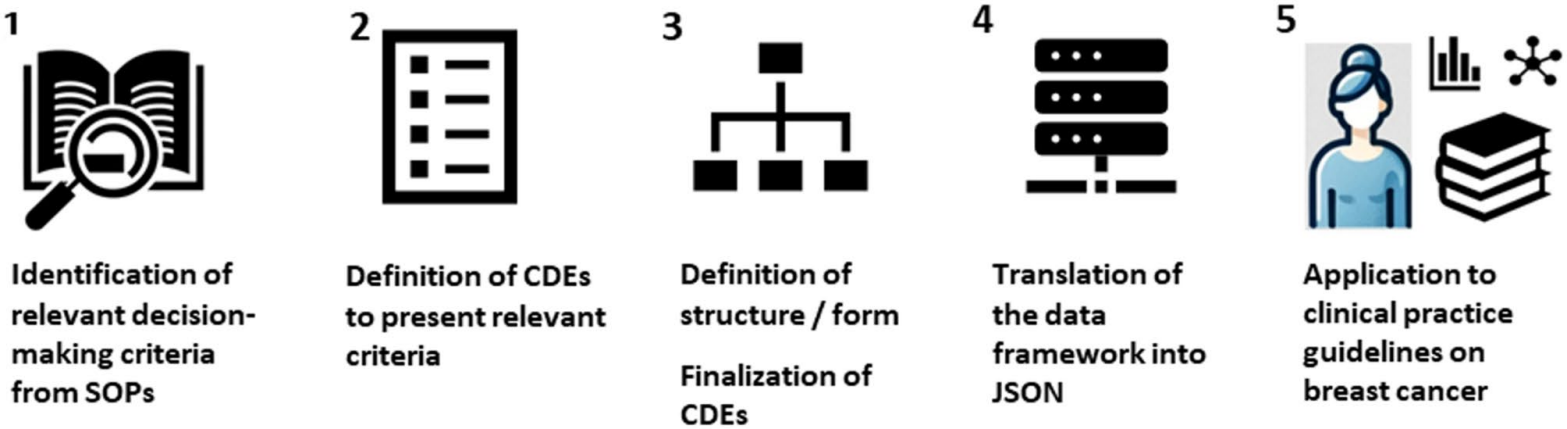
Schematic illustration of the steps used in the methodology for creating and applying the defined CDE-based data structure for radiotherapeutic decision-making in breast cancer

To create and apply the data structure the following steps were conducted. A schematic overview is presented in Fig. 1.

#### Step 1 - Identification of criteria

The SOPs on radiotherapy in breast cancer patients of the Kantonsspital St. Gallen, which are used for decision-making in clinical practice, were thoroughly analyzed. FD and PMP, who use these SOPs in clinical practice, reviewed the documents to identify and formalize criteria involved in different oncological situations in the decision-making process. The physicians discussed the criteria named in the SOPs and confirmed that these are essential for everyday decision-making in the clinical routine. A list of defined criteria was collected.

#### Step 2 - Definition of corresponding CDEs

The criteria were translated into a formalized structure by defining corresponding CDEs. A defined representation of each criterion was created, consisting of one or several “Value List”- or “Number”-CDEs. Regarding “Value List”-CDEs, the list of “Permissible Values” for each CDE were defined, while regarding “Number”-CDEs, corresponding units were defined. The individual CDEs were iteratively revised and refined by FD, MS and NC until consensus was reached and all ambiguities had been resolved. The set of CDEs was confirmed by all three researchers.

#### Step 3 - Definition of structure / form and finalization of CDEs

While CDEs are basically questions that are to be answered for a certain situation, these questions are being asked in a defined setting and regarding a defined concept. As for example, for the concept “breast cancer disease”, a CDE “type of conducted surgery” may be defined. Formulating it as a question, it would mean “What type of surgery was conducted for the breast cancer disease?”.

A “breast cancer disease” may consist of one or multiple “tumor lesions” (e.g., satellite lesions or metastases). In this scenario, a CDE like “tumor size” would make sense on the level of the concept “tumor lesion” but not on the level of “breast cancer disease” (“What size is the tumor lesion?” and not “What size is the breast cancer disease?”). The CDEs were therefore assigned to concepts within the data structure where they should be applied. A hierarchical structure was thereby created. While this structuring is not per se inherent to the CDEs as broadly defined by the NIH, a corresponding delineation is realized with CDE forms [21]. It should overall be noted that the structuring of data in the clinical domain using hierarchies or graphs is a well-established approach in other forms of data standards such as e.g., SNOMED [22].

The structuring and finalization were done by FD, MS and NC and again iteratively revised until consensus was reached.

#### Step 4 – Translation of the drafted data framework into a machine-readable format

To realize a determined and computer-readable format, the structure was translated into the JavaScript Object Notation (JSON) format.

A simplified JSON object encompassing the structure was created. Since JSON is also used by the NIH for defining CDEs, our JSON object was created in analogy to this.

Individual oncological situations can then be presented using the provided JSON object by replacing the CDE concept descriptions with the corresponding values of the situation.

#### Step 5 – Application of the data framework to ASTRO practice guidelines for radiotherapeutic decision-making in breast cancer patients

After completion of the data structure, it would be ready to be used for the presentation of individual breast cancer situations and application for radiotherapeutic decision-making as defined within the local SOPs. To investigate to what extent this data structure could also be used for radiotherapeutic decision-making outside of the local environment, it was applied to the clinical practice guidelines for breast cancer of the American Society for Radiation Oncology (ASTRO).

The ASTRO currently (state April 2025) has published six clinical practice guidelines on breast cancer, covering different specific areas [23].

Namely, these are:

- The ASTRO Guideline on Partial Breast Irradiation for Patients With Early-Stage Invasive Breast Cancer or Ductal Carcinoma In Situ, published in 2023 **(PBI-Guideline)** [24].
- The consensus guideline of the American Society of Clinical Oncology (ASCO), the ASTRO and the Society of Surgical Oncology (SSO) on the Management of Hereditary Breast Cancer, published in 2020 **(Hereditary Breast Cancer-Guideline)** [25].
- The ASTRO Evidence-Based Guideline on Radiation Therapy for the Whole Breast, published in 2018 **(WBI-Guideline)** [26].
- The SSO/ASTRO/ASCO Consensus Guideline on Margins for Breast Conserving Surgery with Whole Breast Irradiation in Ductal Carcinoma in Situ (DCIS), published in 2016 **(Margins-DCIS-Guideline)** [27].
- The ASCO/ASTRO/SSO Postmastectomy Radiotherapy Guideline, first published in 2001 and updated in 2016 **(PMRT-Guideline)** [28].
- The SSO/ASTRO Consensus Guideline on Margins for Breast-Conserving Surgery with Whole-Breast Irradiation in Stages I and II Invasive Breast Cancer, published in 2014 **(Margins-BC-Guideline)** [29].

These guidelines and consensus papers contain valuable recommendations for practical radiotherapeutic decision-making in treating breast cancer patients. Similar to the local SOPs, the recommendations also contain various criteria the individual decision is based on.

All the recommendations and mentioned criteria of the six practice guidelines were analyzed in full text to identify all mentionings of some sort of decision-making criteria in the individual guideline recommendations. For each recommendation, two researchers (FD and NC) independently listed all the decision-making criteria mentioned. These two lists were merged, and disagreements were discussed among the two researchers. For the final list of decision-making criteria for each recommendation, it was discussed if the criterion is presentable with the data structure (yes or no) and it was defined which CDEs in the data structure would be needed therefore. A third researcher (PMP) was available for making a final decision in cases of persistent disagreement. Finally it was checked whether the entire situation (meaning all listed decision-making criteria) could be presented with the data structure.

## Results

### Criteria and CDEs for radiotherapeutic decision-making in breast cancer patients

In step 1 a total of 31 different criteria that are involved in radiotherapeutic decision-making for breast cancer patients were identified when analyzing the SOP documents. These involved general person-related criteria, criteria regarding tumor location, criteria related to cancer stage, histopathological and genetic criteria as well as criteria about previously conducted oncological therapies.

In step 2 a total of 46 CDEs were defined to present these criteria. These included 36 “Value List”-CDEs and 10 “Number”-CDEs. There were no CDEs of the other data types defined by the NIH (being “Text”, “Date”, “File” and “Externally Defined”). For 18 of the criteria exactly one corresponding CDE was defined. In 12 cases, two CDEs were defined to describe the criterion. For one criterion (= tumor size), four corresponding CDEs were defined.

A basic overview of the criteria and the corresponding CDEs used to describe them is provided in Table 1. More detailed information and description of the CDEs is provided in Appendix 1.

**Table 1:** List of identified criteria for radiotherapeutic decision-making in breast cancer patients together with CDEs defined to represent them

	Name of criterion	Name of CDE	Type of CDE
General person-related information			
	Age	Age	Number
	Menopausal Status	Menopausal Status	Value List
Tumor location			
	Laterality	Laterality	Value List
	**Tumor location**	Tumor location	Value List
Cancer stage			
	cT-Stage	cT-Stage Primary or Recurrent (cT)	Value List Value List
	pT-Stage	pT-Stage Primary or Recurrent (pT)	Value List Value List
	cN-Stage	cN-Stage Primary or Recurrent (cN)	Value List Value List
	pN-Stage	pN-Stage Primary or Recurrent (cN)	Value List Value List
	cM-Stage	cM-Stage Primary or Recurrent (cM)	Value List Value List
	pM-Stage	pM-Stage Primary or Recurrent (pM)	Value List Value List
	Sentinel lymph node status	sentinel lymph node status	Value List
	Lymph node involvement in the mammaria interna region	Lymph node involvement in the mammaria interna region	Value List
	Invasive carcinoma with associated DCIS	Primary or Recurrent associated DCIS of other tumor lesion	Value List Value List
Further histopathological and genetic criteria			
	G status	G status	Value List
	L status	L status	Value List
	V status	V status	Value List
	R status	R status	Value List
	Her2-receptor status	Her2-receptor status (IHC) Her2-receptor status (positive/negative)	Value List Value List
	Histological subtype	Histological subtype	Value List
	Extracapsular extension of a lymph node metastasis	Extracapsular extension of a lymph node metastasis	Value List
	BRCA status	BRCA1-Status BRCA2-Status	Value List Value List
	Minimal resection margin	Minimal resection margin	Number
	Tumor size	Diameter1 Diameter2 Diameter3 Modality of Assessment	Number Number Number ValueList
	Number of positive resected lymph nodes	Number of positive resected lymph nodes	Number
	Number of resected lymph nodes	Number of resected lymph nodes	Number
	Estrogen receptor expression	ER status (%) ER status (positive/negative)	Number Value List
	Progesterone receptor expression	PR status (%) PR status (positive/negative)	Number Value List
	Ki-67	Ki-67 status (%)	Number
Previously conducted oncological therapies			
	Conduction of neoadjuvant chemotherapy	Conduction of neoadjuvant chemotherapy	Value List
	Post-resection performed	Post-resection performed	Value List
	Previous surgery	Previous surgery conducted Type of previously conducted surgery	Value List Value List

### Conceptualization of the data structure

**Fig. 2:**
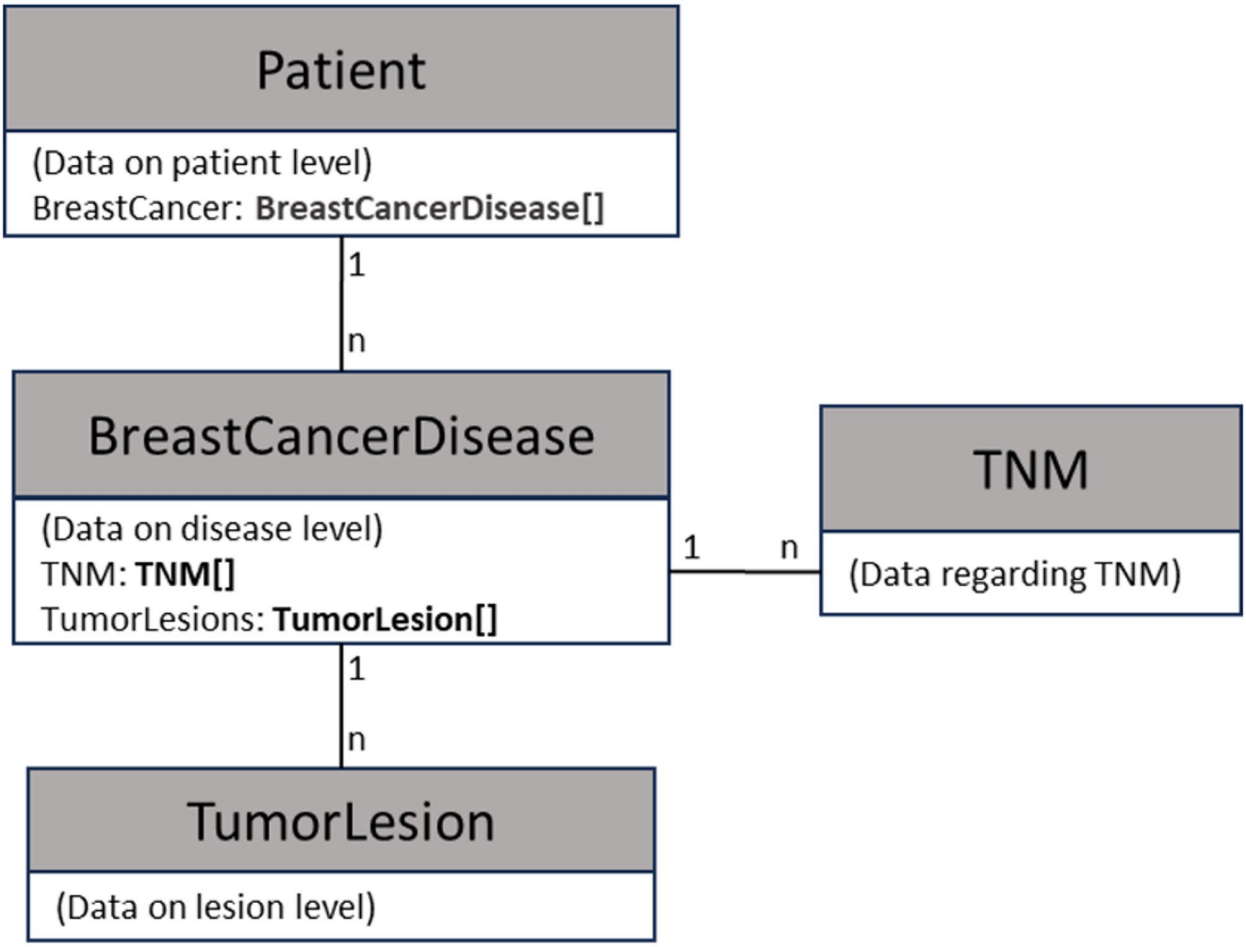
Schematic illustration of the four main concepts (= classes) and relationships used in the data structure

In step 3 a hierarchy with presentation of the CDEs in an object-oriented structure was created. Four main classes, representing four conceptual levels, within which CDEs are used, were defined, namely “Patient”, “BreastCancerDisease”, “TNM” (= Classification system to stage the cancer situation based on Tumor, Lymph-Node and Metastasis) and “TumorLesion” (Fig. 2).

The class “Patient” has the attribute BreastCancer, which is an array of objects of type “BreastCancerDisease”. “BreastCancerDisease” has the attributes TNM (Array of type TNM) and TumorLesions (Array of type TumorLesion). The rationale of this structuring is the following: A patient can have zero, one or more than one independent breast cancer diseases. A breast cancer disease consists of one or several tumor lesions (including recurrent lesion, associated DCIS, satellite lesion or metastases; lymph node metastases are handled separately). Each breast cancer disease can be staged according to TNM (it should be noted that there exist several TNM classification systems, most notably according to UICC and to AJCC; it should be clearly defined, which system is use – in our study we used the UICC system; see also Appendix 1). There can be several TNM stagings of a disease, including differences regarding “clinical” and “pathological” staging as well as stagings happening at different time points, that may all be relevant for clinical decision-making.

**Fig. 3:**
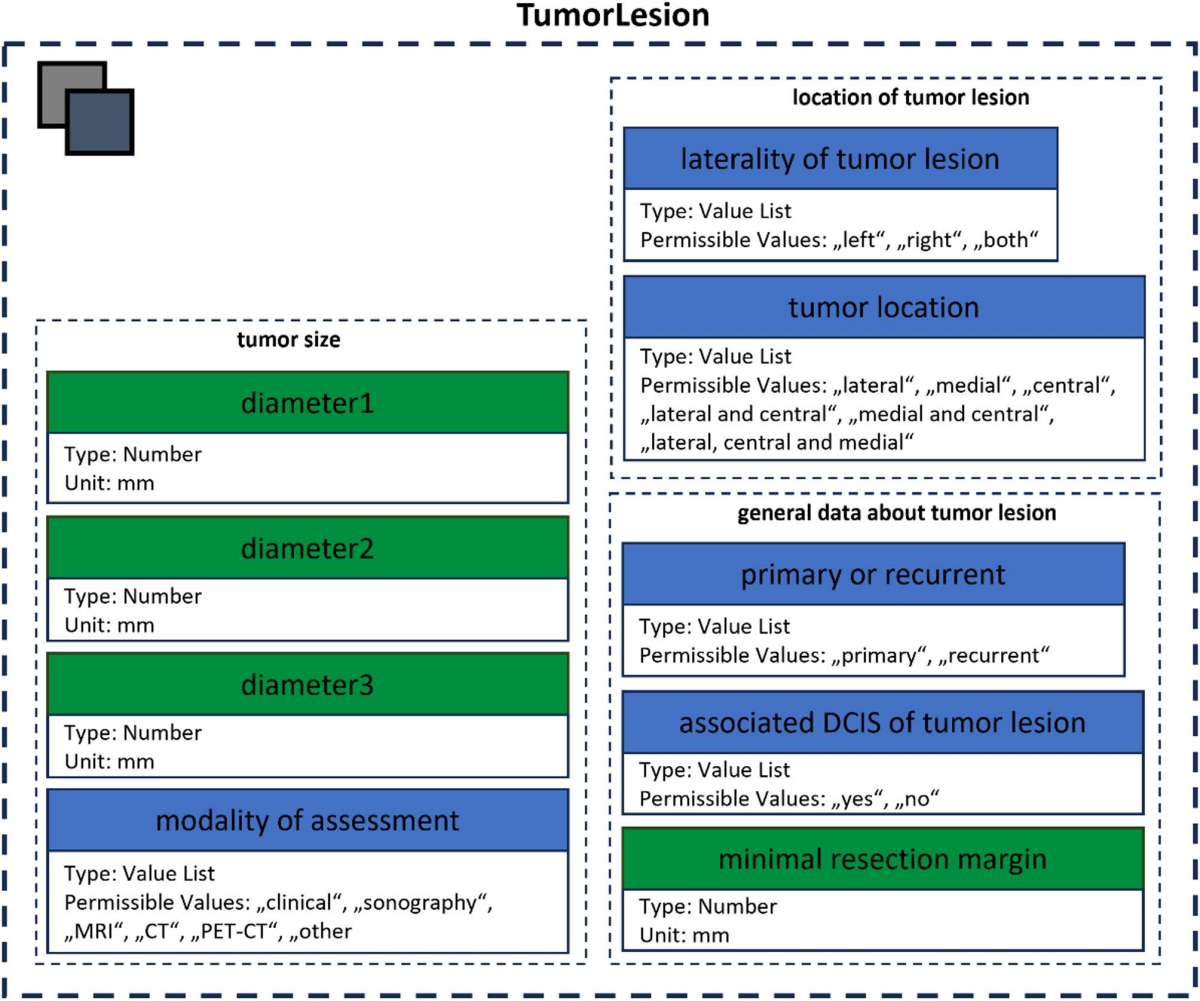
Structure of the class “TumorLesion”, which contains the subclasses “location of tumor lesion”, “general data about tumor lesion” and “tumor size”. “Value List”-CDEs are presented in blue, “Number”-CDEs in green

Further subclasses for organizing and structuring of the CDEs were defined – an example for “TumorLesion” with the subclasses “location of tumor lesion”, “general data about tumor lesion” and “tumor size” is provided in Fig. 3.

The overall structure as a diagram is provided in Appendix 2 while a list with the parameters of the CDEs and further descriptions is provided in Appendix 1.

All the subclasses have a 1:1 relationship between parent class and subclass. While they are helpful for improved overview when organizing the CDEs, they are not necessary from a semantical point of view and the CDEs could also be defined as direct attributes in the main classes.

In step 4 a corresponding JSON format encompassing the three main classes, subclasses and CDEs was created. The final structure that can be applied to various breast cancer situations, is provided in Appendix 3.

### Application of the data framework to ASTRO practice guidelines

In step 5 the six mentioned clinical practice guidelines for breast cancer published by the ASTRO were analyzed in full text. A total of 94 recommendations were identified across the guidelines with 90 recommendations mentioning some sort of decision-making criteria. After initial listing of the decision-making criteria in all but three cases the two researchers had identified the same criteria. In these three cases of disagreement consensus among the two researchers was reached without persistent disagreement, leading to a finalized list of 216 decision-making criteria. In 151 cases (70.0%), the criterion could be presented using the data structure, while in the other 65 cases the required semantic information could not fully be described with the data structure. Consensus among the two researchers was reached in all cases without persistent disagreement. For 52 of the guideline recommendations (not including the four recommendations that did not mention any decision-making criteria), all mentioned criteria of the recommendation were fully presentable using the data structure (57.7%).

**Fig. 4:**
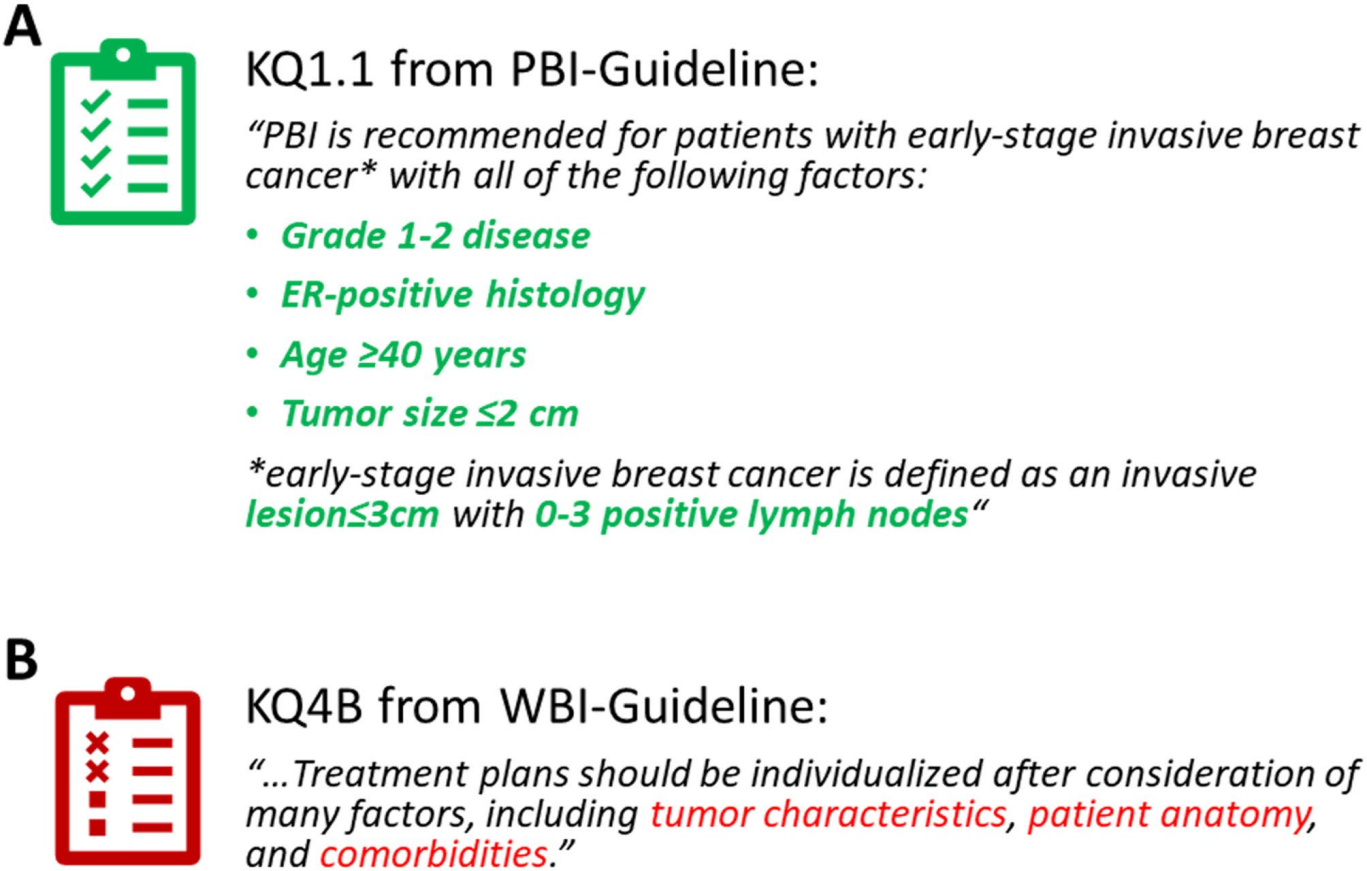
Example of two recommendations from the guidelines that can either be (**A**) fully presented using the data structure with all mentioned decision-making criteria implemented in the CDE-based data structure or (**B**) not presentable using the data structure since some of the relevant information involved in the decision-making is not implemented

An example of a fully presentable recommendation as well as one example of a not presentable recommendation is illustrated in Fig. 4.

The portion of presentable criteria and the amount of guideline recommendations that could be fully presented varied among the six guidelines (see Table 2).

**Table 2:** Amount of presentable recommendations and criteria in the six clinical practice guidelines of the ASTRO

Practice Guideline	Presentable criteria / total number of criteria	Presentable recommendations / total number of recommendations
PBI-Guideline	53/56 (94.6%)	17/20 (85.0%)
Hereditary Breast Cancer-Guideline	25/52 (48.1%)	8/22 (36.4%)
WBI-Guideline	35/56 (62.5%)	12/27 (44.4%)
Margins for DCIS-Guideline	10/11 (90.1%)	7/8 (87.5%)
PMRT-Guideline	18/26 (69.2%)	4/6 (66.7%)
Margins for Breast Cancer-Guideline	10/15 (66.7%)	4/7 (57.1%)

The data with the individual criteria mentioned in the recommendations of the six guidelines and the CDEs of the data structured used to present them is provided in Appendix 4.

## Discussion

### CDE-based structuring of data for radiotherapeutic Decision-Making on the level of a local hospital

We successfully developed a data structure based on local SOPS on radiotherapeutic decision-making in breast cancer, which is machine-readable and can present 31 relevant decision-making criteria using 46 CDEs. Applied to six clinical practice guidelines of the ASTRO, relevant mentioned decision-making criteria could be presented in 151 of 216 cases.

For comparison, Mirbagheri et al. who used the CDE concept to develop a minimum data set for a breast cancer registry system in Iran, created a system with 205 CDEs [30]. It is not surprising that they defined many more CDEs since within the registry a lot of information is collected that is not considered relevant for clinical decision-making in our SOPs (e.g., socioeconomic data, care facility information, legal data, etc.). While the data collection for cancer registries and clinical trials is much more comprehensive than for routine clinical practice, we can still effectively implement CDEs - standardized, clearly defined questions and data points - across these different contexts. Answering > 200 questions about a real-world breast cancer case would be very cumbersome. However, by answering 46 questions we can cover all recommendations in our local SOPs and 70% of recommendations in ASTRO guidelines on breast cancer. It may still not seem practical for a clinician to answer that many question – however, as we have seen in one of our recent studies, modern systems for Natural Language Processing (NLP) including generative AI, such as Large Language Models (LLMs) can automatically answer/extract dozens of CDEs from clinical documents with high levels accuracy in a matter of a few seconds [31]. As generative AI gets increasingly implemented in healthcare many laborious tasks for data documentation that traditionally required human-level understanding and reasoning can be automated [32]. As a result, clear data concepts facilitating interoperability become even more important.

Nevertheless, the implementation of a CDE-based system for data collection in a local hospital setting requires a considerable amount of planning, training, and integration with existing concepts, standards and healthcare IT systems. However, the payoff in terms of improved data quality, enhanced monitoring of cancer situations, and streamlined clinical workflows could be substantial [33].

In the dynamic and nuanced field of radiotherapy for breast cancer patients, the utilization of CDEs marks a transformative shift towards personalized and evidence-based treatment. By implementing a CDE-based framework, healthcare professionals could access a consolidated view of a patient’s comprehensive medical history and current health status, enabling the formulation of a tailored radiotherapeutic strategy.

### Leveraging information exchange up to the level of clinical practice guidelines

The data structure in our work was created as a first concept to formalize decision-making criteria used on the level of our local hospital. Yet, we have seen that a considerable amount of the criteria mentioned in international clinical practice guidelines can also be presented using this structure (ranging from 48.1 to 94.6%; see Table 2). This may not be surprising, since local SOPs should align with the recommendations of established clinical practice guidelines. One may assume that the relevant decision-making criteria are shared on a broader level among different facilities. However, it has been repeatedly shown for many examples in oncological decision-making, that this assumption is clearly wrong [7, 34, 35]. As we had also recently seen in a document analysis study that aimed to find shared CDEs among radiotherapy departments when ordering a planning CT, the majority of data cannot be exchanged [11]. Overall, exchange of standardized data is often very limited among different facilities. Neither the local SOPs, nor the six ASTRO guidelines were developed with a focus on interoperability or clear definition and formalization of criteria and concepts. It is not surprising that e.g., for the Hereditary Breast Cancer Guideline, the applicability was limited with presentable criteria at only 48.1% (Table 2). Hereditary breast cancer is a special situation that was not fully addressed in the data structure based on the local SOPs. Nevertheless, the fact that overall, in a majority of 70.0% of cases a mentioned criterion in the guidelines was representable, confirms the usage of CDEs for promoting interoperability.

### A shared CDE-based data structure

The data structure could be extended to cover more of the decision-making criteria mentioned in the ASTRO guidelines to be further applicable. Adjusting the data structure for implementation of international guidelines should be based on broader consensus including clinical experts involved in guideline creation. As the ISROI will continue to work on the structuring of radiotherapeutic data using CDEs, such endeavors will be the subject of future initiatives. In any case, both the medical and informatics perspectives must be maintained to clearly define concepts, criteria and CDEs.

One major advantage of the CDE-based data structure is that it could be used in a modular way without giving up the overall structure itself. For example, one could create an additional subclass for the information regarding hereditary breast cancer to cover the related information. This subclass could be implemented in a more comprehensive version of the data structure and only be used if relevant for a specific question in a certain guideline or local SOP.

If a functioning, well designed data structure is in place, CDEs could also empower downstream systems like integrated CDSS or clinical algorithms to deliver more accurate, timely, and personalized medical advice and treatment recommendations [36]. Since one major goal of CDEs is the promotion of standardization and interoperability, they should not remain abstract concepts but be applied in clinical practice at the local hospital level.

If diverse stakeholders were to adopt a common CDE-based data framework, it would enable local hospitals to engage in collaborative decision-making and enhance standardization and data sharing across institutions. Although such a framework has not yet been realized, its implementation is essential for facilitating data exchange among various institutions and expanding the use of IT and AI solutions on a larger scale.

### Limitations, challenges and possible solutions

Even though the CDE-based data structure developed in this work allows for clear documentation and communication of medical data, the approach itself has some limitations. Theoretically, if the values of all the CDEs of a breast cancer situation are known, any such situation could be unambiguously presented in the proposed JSON format. However, in clinical practice, ambiguities, uncertainties and contradictory data are common. To use the framework on real data encompassing such ambiguities, uncertainties and contradictions, one could define a clear methodology to assign CDE values to real world situations. However, for versatile situations like breast cancer, even very sophisticated methods can not cover every possible situation that may need to be addressed during such value assignment [37]. Yet, this is more a fundamental problem of suboptimal and ambiguous data than of the structuring approach itself.

Nevertheless, the structure in its current form as it is based on the original CDE definition is not all-encompassing. One issue is that the value of any data element is assessed at one certain time point, which is not addressed in the data structure. An obvious example is the CDE “Age”, which changes as the patient gets older. The values are therefore not necessarily static but may have a dynamic that is not implemented currently. One possible solution would be to extend the concept of CDEs within the framework and define the values with an additional (optional) timestamp. In situations where multiple data points for a CDE are provided, the corresponding attribute in the JSON object could be presented with an array of objects containing the value and (if known) the time stamp. A similar idea of such an array-format is in the current structure only provided for the concept “TNM”, as it was considered a relevant dynamic factor in the analyzed SOPs.

A further interesting idea to extend the possibilities of CDE-based data documentation was proposed by Kim et al. [38]. They introduced so-called “composite relationships” and defined the constraints “operated”, “required”, “dependent” and “ordered” in a proposed extension of the CDE concept. Defining such relationships, it is possible to establish a broader semantic framework.

However, even if very complex systems were used, it would be very challenging to depict not only the data of broader, well-established concepts, but also detailed information and nuances of a medical situation. If CDEs are to be used for facilitating interoperability, they need to be implemented on a local level as well as on a broader abstract level. To address individual needs, also the criteria used only by a few actors (so-called insular criteria [39]) would need to be addressed since they may influence local decision-making. Overall, relevant criteria and corresponding CDEs need clear definitions. The reason why many criteria mentioned in the ASTRO guidelines could not be presented with the data structure is due to unclear/missing definitions. Criteria like “tumor characteristics”, “patient anatomy” or “comorbidities” (see Fig. 4B) are certainly relevant, but are not clearly defined within the analyzed guidelines. As a result, these criteria remain ambiguous. Taking the effort of presenting criteria with structures based on CDEs formalizes these criteria and thereby promotes standardization and interoperability while reducing ambiguities. Clearly defining rather abstract criteria may take a considerable amount of effort, but is possible (as for example done for “comorbidities” by the NCI on the US population [40]). It has to be acknowledged however, that not every possible criterion can be formalized using the CDE concept (e.g., a criterion like “high risk of complications“). Presenting medical information with CDEs resembles the creation of a synoptic report, which gives a concise and clear overview, but a priori is a simplification.

The data structure in this work is not an all-encompassing framework that could be used on a broader inter-institutional level to collect all relevant information for radiotherapeutic decision-making. Current definitions are also not truly unambiguous, so that all possible situations and questions can be answered (see also further information on the CDEs in Appendix 1). The CDEs, classes and subclasses of the data structure will need to be extended, refined and aligned with existing medical data standards in the future. However, it may be a starting point originating from real world clinical decision-making. It can build a basis for further initiatives conducted by the ISROI and other stakeholders to promote structuring and formalization of relevant data and criteria used in clinical practice for radiation therapy and general oncology.

The study itself has some limitations. While multiple researchers designed and iteratively refined the framework both from a medical and a data science perspective, the used methodology is to some extent subjective. We did not use more rigorous scientific methods such as systematic reviews or Delphi surveys in identifying and evaluating CDEs for this case study. The way we defined and evaluated the data structure may be biased. No systematic search alignment with existing CDE resources has been conducted. Overall, more sophisticated and elaborate methods like Delphi rounds among independent researchers will be needed, if a standardized data structure is to be established for documentation and exchange of data across different stakeholders on an inter-institutional level.

## Conclusions

The development of a CDE-based data structure for radiotherapy in breast cancer demonstrates a practical and scalable approach to structure medical data organization. By translating clinical decision-making criteria from local SOPs into clearly defined CDEs, this study establishes a structure, machine-readable framework for enhanced data interoperability and integration with IT/AI systems. At the same time, the application of the data framework to clinical practice guidelines showcases the potential for broader adoption. While limitations exist—such as ambiguities in real-world data and the need for further refinement—a CDE-based approach can lay the groundwork for semantic interoperability across institutions. Future efforts will focus on expanding and refining CDEs through multidisciplinary consensus, aligning with existing standards, and addressing dynamic data needs.

## Electronic supplementary material

Below is the link to the electronic supplementary material.

Supplementary Material 1

Supplementary Material 2

Supplementary Material 3

Supplementary Material 4

## Data Availability

All the data obtained in the study (created CDEs and structures) are provided in this work or its Supplementary Material. The six ASTRO guidelines have previously been published with the publications listed in the References [12, [17].

## Abbreviations

AI: Artificial intelligence
AJCC: American Joint Committee on Cancer
ASCO: American Society of Clinical Oncology
ASTRO: American Society for Radiation Oncology
BOLD: Breast Oncology Local Disease
CDE: Common Data Element
CDSS: Clinical Decision Support System
CIS: Clinical Information System
CRF: Case Report form
EHR: Electronic Health Record
ISROI: International Society for Radiation Oncology
IT: Information technology
JSON: JavaScript Object Notation
Hereditary Breast Cancer: Guildeline–ASTRO/ASCO/SSO Consensus guideline on the Management of Hereditary Breast Cancer
Margins: BC–Guideline–SSO/ASTRO Consensus Guideline on Margins for Breast–Conserving Surgery with Whole–Breast Irradiation in Stages I and II Invasive Breast Cancer
Margins: DCIS–Guideline–SSO/ASTRO/ASCO Consensus Guideline on Margins for Breast Conserving Surgery with Whole Breast Irradiation in DCIS
NCI: National Cancer Institute
NIH: National Institutes of Health
NLP: Natural Language Processing
PBI: Guideline–ASTRO Guideline on Partial Breast Irradiation for Patients With Early–Stage Invasive Breast Cancer or Ductal Carcinoma In Situ
PMRT: Guideline–ASCO/ASTRO/SSO Postmastectomy Radiotherapy Guideline
RO: Radiation Oncology
RWD: Real–world data
SSO: Society of Surgical Oncology
SOP: Standard operating procedure
UICC: Union for International Cancer Control
WBI: Guideline–ASTRO Evidence–Based Guideline on Radiation Therapy for the Whole Breast
