# Supplementary material for: A CDE-based data structure for radiotherapeutic decision-making in breast cancer
Source: BMC Med Inform Decis Mak. 2025 Jul 1;25:220. doi: 10.1186/s12911-025-03036-1 (PMC12211407; doi:10.1186/s12911-025-03036-1)
Supplement: Supplementary file 2 — Supplementary Material 2 [file 12911_2025_3036_MOESM2_ESM.pdf]

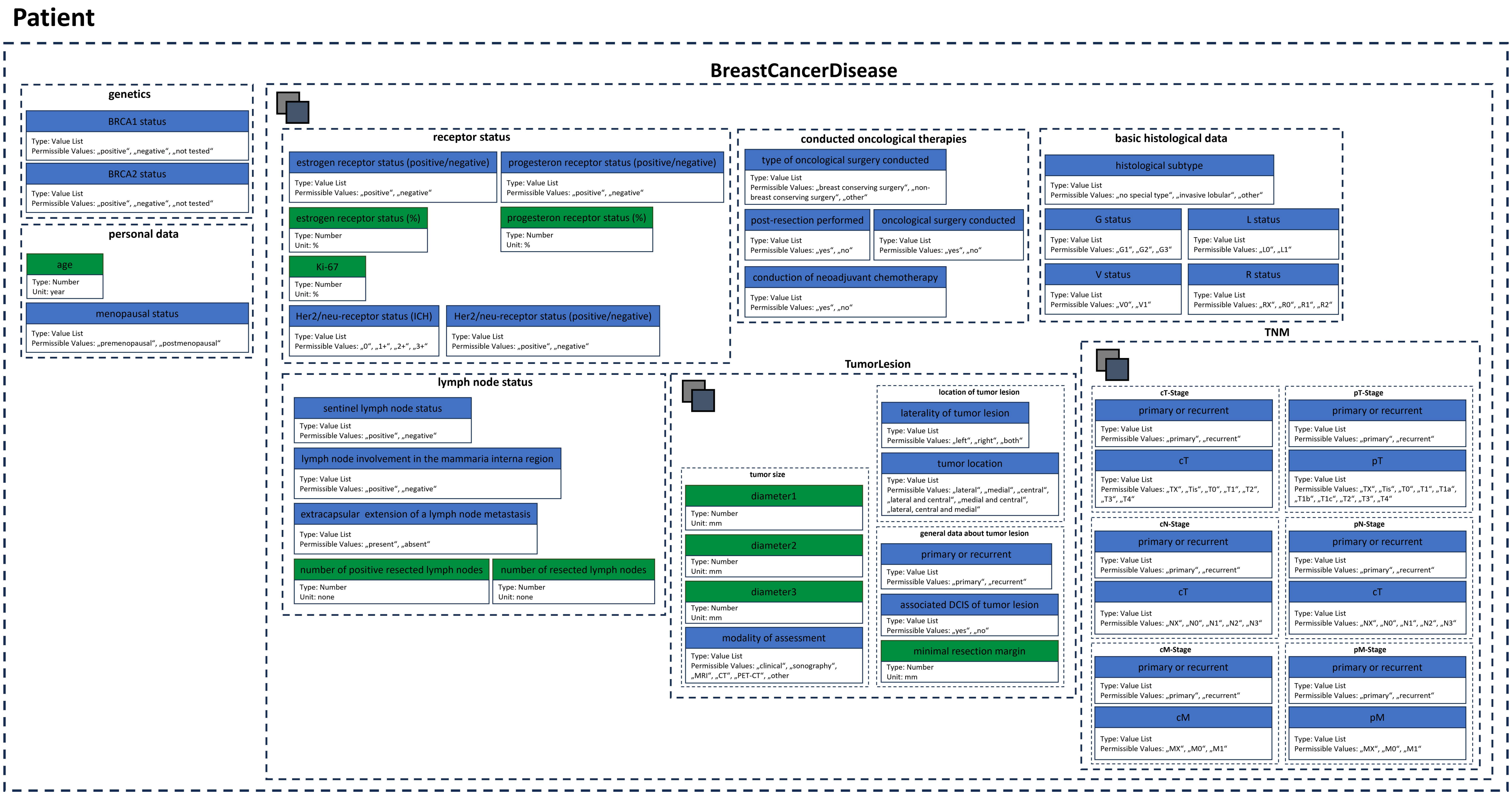

Patient
BreastCancerDisease
genetics
BRCA1 status
Type: Value List
Permissible Values: „positive“, „negative“, „not tested“
BRCA2 status
Type: Value List
Permissible Values: „positive“, „negative“, „not tested“
personal data
age
Type: Number
Unit: year
menopausal status
Type: Value List
Permissible Values: „premenopausal“, „postmenopausal“
receptor status
estrogen receptor status (positive/negative)
Type: Value List
Permissible Values: „positive“, „negative“
progesteron receptor status (positive/negative)
Type: Value List
Permissible Values: „positive“, „negative“
estrogen receptor status (%)
Type: Number
Unit: %
progesteron receptor status (%)
Type: Number
Unit: %
Ki-67
Type: Number
Unit: %
Her2/neu-receptor status (ICH)
Type: Value List
Permissible Values: „0“, „1+“, „2+“, „3+“
Her2/neu-receptor status (positive/negative)
Type: Value List
Permissible Values: „positive“, „negative“
conducted oncological therapies
type of oncological surgery conducted
Type: Value List
Permissible Values: „breast conserving surgery“, „non-breast conserving surgery“, „other“
post-resection performed
Type: Value List
Permissible Values: „yes“, „no“
oncological surgery conducted
Type: Value List
Permissible Values: „yes“, „no“
conduction of neoadjuvant chemotherapy
Type: Value List
Permissible Values: „yes“, „no“
basic histological data
histological subtype
Type: Value List
Permissible Values: „no special type“, „invasive lobular“, „other“
G status
Type: Value List
Permissible Values: „G1“, „G2“, „G3“
L status
Type: Value List
Permissible Values: „L0“, „L1“
V status
Type: Value List
Permissible Values: „V0“, „V1“
R status
Type: Value List
Permissible Values: „RX“, „R0“, „R1“, „R2“
lymph node status
sentinel lymph node status
Type: Value List
Permissible Values: „positive“, „negative“
lymph node involvement in the mammaria interna region
Type: Value List
Permissible Values: „positive“, „negative“
extracapsular extension of a lymph node metastasis
Type: Value List
Permissible Values: „present“, „absent“
number of positive resected lymph nodes
Type: Number
Unit: none
number of resected lymph nodes
Type: Number
Unit: none
TumorLesion
tumor size
diameter1
Type: Number
Unit: mm
diameter2
Type: Number
Unit: mm
diameter3
Type: Number
Unit: mm
modality of assessment
Type: Value List
Permissible Values: „clinical“, „sonography“, „MRI“, „CT“, „PET-CT“, „other
location of tumor lesion
laterality of tumor lesion
Type: Value List
Permissible Values: „left“, „right“, „both“
tumor location
Type: Value List
Permissible Values: „lateral“, „medial“, „central“, „lateral and central“, „medial and central“, „lateral, central and medial“
general data about tumor lesion
primary or recurrent
Type: Value List
Permissible Values: „primary“, „recurrent“
associated DCIS of tumor lesion
Type: Value List
Permissible Values: „yes“, „no“
minimal resection margin
Type: Number
Unit: mm
TNM
cT-Stage
primary or recurrent
Type: Value List
Permissible Values: „primary“, „recurrent“
cT
Type: Value List
Permissible Values: „TX“, „Tis“, „T0“, „T1“, „T2“, „T3“, „T4“
pT-Stage
primary or recurrent
Type: Value List
Permissible Values: „primary“, „recurrent“
pT
Type: Value List
Permissible Values: „TX“, „Tis“, „T0“, „T1“, „T1a“, „T1b“, „T1c“, „T2“, „T3“, „T4“
cN-Stage
primary or recurrent
Type: Value List
Permissible Values: „primary“, „recurrent“
cT
Type: Value List
Permissible Values: „NX“, „N0“, „N1“, „N2“, „N3“
pN-Stage
primary or recurrent
Type: Value List
Permissible Values: „primary“, „recurrent“
cT
Type: Value List
Permissible Values: „NX“, „N0“, „N1“, „N2“, „N3“
cM-Stage
primary or recurrent
Type: Value List
Permissible Values: „primary“, „recurrent“
cM
Type: Value List
Permissible Values: „MX“, „M0“, „M1“
pM-Stage
primary or recurrent
Type: Value List
Permissible Values: „primary“, „recurrent“
pM
Type: Value List
Permissible Values: „MX“, „M0“, „M1“
